# Gut Microbiome-Mediated Mechanisms in Alleviating Opioid Addiction with Aqueous Extract of *Anacyclus pyrethrum*

**DOI:** 10.3390/biomedicines12061152

**Published:** 2024-05-23

**Authors:** Abdelmounaim Baslam, Hamid Kabdy, Yassine Chait, Hajar Azraida, Loubna El Yazouli, Rachida Aboufatima, Abderrahman Chait, Marouane Baslam

**Affiliations:** 1 Laboratory of Pharmacology, Neurobiology, Anthropobiology and Environment, Department of Biology, Faculty of Sciences Semlalia, Cadi Ayyad University, Marrakech 40000, Morocco; baslamounaim@gmail.com (A.B.); hajarazraida@gmail.com (H.A.);; 2 Agadir Souss Massa University Hospital, Faculty of Medicine and Pharmacy, Ibn Zohr University, Agadir 80000, Morocco; 3 Laboratory of Biological Engineering, Faculty of Sciences and Technology, Sultan Moulay Slimane University, Beni Mellal 23000, Morocco; 4 Center of Agrobiotechnology and Bioengineering, Research Unit Labelled CNRST (Centre AgroBiotech-URL-7 CNRST-05), Cadi Ayyad University, Marrakech 40000, Morocco; 5 Laboratory of Agro-Food, Biotechnologies and Valorization of Plant Bioresources (AGROBIOVAL), Department of Biology, Faculty of Science Semlalia, Cadi Ayyad University (UCA), Marrakech 40000, Morocco; 6 Laboratory of Biochemistry, Department of Applied Biological Chemistry, Faculty of Agriculture, University of Niigata, Niigata 950-2181, Japan; 7 GrowSmart, Seoul 07516, Republic of Korea

**Keywords:** addiction, drug dependence, opioid use disorder (OUD), fentanyl withdrawal, gut microbiota, oxidative stress, *Anacyclus pyrethrum*, therapeutic potential, substance abuse

## Abstract

The escalating rates of morbidity and mortality associated with opioid use disorder (OUD) have spurred a critical need for improved treatment outcomes. This study aimed to investigate the impact of prolonged exposure to Fentanyl, a potent opioid, on behavior, biochemical markers, oxidative stress, and the composition of the gut microbiome. Additionally, we sought to explore the therapeutic potential of *Anacyclus pyrethrum* in mitigating the adverse effects of Fentanyl withdrawal. The study unveiled that chronic Fentanyl administration induced a withdrawal syndrome characterized by elevated cortisol levels (12.09 mg/mL, compared to 6.3 mg/mL for the control group). This was accompanied by heightened anxiety, indicated by a reduction in time spent and entries made into the open arm in the Elevated Plus Maze Test, as well as depressive-like behaviors, manifested through increased immobility time in the Forced Swim Test. Additionally, Fentanyl exposure correlated with decreased gut microbiome density and diversity, coupled with heightened oxidative stress levels, evidenced by elevated malondialdehyde (MDA) and reduced levels of catalase (CAT) and superoxide dismutase (SOD). However, both post- and co-administration of *A. pyrethrum* exhibited substantial improvements in these adverse effects, effectively alleviating symptoms associated with OUD withdrawal syndrome and eliciting positive influences on gut microbiota. In conclusion, this research underscores the therapeutic potential of *A. pyrethrum* in managing Fentanyl withdrawal symptoms. The findings indicate promising effects in alleviating behavioral impairments, reducing stress, restoring gut microbiota, and mitigating oxidative stress, offering valuable insights for addressing the challenges of OUD treatment.

## 1. Introduction

Substance use disorders (SUD) represent a significant global health challenge, characterized by chronic neuropsychiatric manifestations that result in difficulties regulating drug consumption and adverse emotional states upon drug unavailability [1]. The pervasive impact of SUD is evident in its contribution to high disability rates, premature deaths, and substantial economic burdens on the healthcare, law enforcement, and productivity sectors [2]. With approximately 300 million individuals worldwide using psychoactive substances and 39.5 million suffering from SUD, the need for effective treatment interventions is paramount [United Nations Office on Drugs and Crime, UNODC] [3]. Opioids, particularly Fentanyl, have emerged as a major concern, impacting a significant portion of the global population (3.8%) and posing serious public health risks [4]. Fentanyl’s potent activation of μ-opioid receptors induces intense euphoria, sedation, and relaxation, contributing to its widespread use and associated health consequences [5].

The neurobiological effects of addictive substances, including the elevation of dopamine (DA) release within the nucleus accumbens, play a pivotal role in the pleasurable effects of substance (ab)use [6,7]. Moreover, SUD contribute to the amplification of oxidative stress in the brain, primarily through rapid oxidation of DA and enzymatic metabolism, leading to the production of reactive oxygen species (ROS) [8]. In fact, SUD trigger the release of DA, which undergoes rapid spontaneous oxidation or is metabolized by enzymes, including monoamine oxidase B (MAO-B) and cyclooxygenase 1/2 (COX1/2). This process leads to the production of superoxide ions and hydrogen peroxide, thereby amplifying oxidative stress in the brain [8]. Additionally, emerging research underscores the bidirectional communication between the gut microbiota (GM) and the brain, highlighting the potential influence of GM composition on neurotransmitter production, which leads to mental health disorders such as anxiety, depression, and stress-related conditions [9,10]. Microbial metabolites produced in the gut can cross the blood–brain barrier and influence brain function [9,10]. The reduction of specific beneficial bacteria can impact the production of neurotransmitters such as GABA [11], glutamate [12], acetylcholine [13], dopamine [14], and serotonin [15]. These neurotransmitters play pivotal roles in influencing mood and behavior [16]. Specific strains of bacteria or their metabolites have been demonstrated to modulate neurotransmitter levels and impact the development and function of the central nervous system. This intricate gut–brain connection emphasizes the significance of microbiome alterations in regulating mood and behavior [10].

Considering the limitations of current treatment modalities for opioid use disorder (OUD), particularly the drawbacks of medication-assisted treatments (i.e., opioid replacement therapy and gradual dosage tapering), there is a pressing need to explore alternative approaches that address these concerns more effectively. Potential side effects, relapse susceptibility, and the requirement for prolonged maintenance pose significant concerns [17]. Understanding the gut–brain axis could offer valuable insights into refining existing treatments and developing novel OUD interventions. In this context, natural products, such as *Anacyclus pyrethrum*, present a promising avenue for discovering new chemical substances with potential therapeutic applications, given their historical use and bioactivity [18,19].

The ethnopharmacology approach provides a valuable framework for drug development against substance abuse, as it leverages the traditional use of medicinal plants by human communities to identify bioactive compounds with potential anti-addictive effects [20]. *Anacyclus pyrethrum* (L.), commonly known by vernacular names such as pellitory, Aqar Qarha, Oud El Attas, and tigandizt, is a member of the Asteraceae family and the Anacyclus genus. Indigenous to regions spanning Morocco, Spain, and Algeria, this wild flowering plant flourishes in arid, rocky terrains, especially in mountainous landscapes [21]. *A. pyrethrum*, known for its diverse phytochemical components, presents an intriguing subject for investigation in the context of OUD. Prior research revealed that *A. pyrethrum* reduced withdrawal states during addiction across different psychoactive substances, such as cigarettes, ecstasy, clonazepam, and trihexyphenidyl, promising the potential modulation of the neuronal pathways associated with SUD [20,22]. The plant’s phytochemical constituents, including alkaloids, flavonoids, and terpenoids, exhibit diverse biological activities that could potentially modulate neuronal pathways involved in SUD.

This research explores the novel relationship between prolonged Fentanyl exposure, a strong opioid, and its impact on gut microbiota and oxidative stress. Additionally, it investigates the potential therapeutic effects of *A. pyrethrum* in alleviating the adverse effects experienced during withdrawal.

## 2. Materials and Methods

### 2.1. Plant Material and Preparation of the Aqueous Extract

The roots of *A. pyrethrum* were assembled from Bin El Ouidan, Morocco (32°7′48″ latitude N/6°27′36″ Longitude W) and authenticated at the laboratory of Pharmacology, Cadi Ayyad University, Marrakech. The voucher specimen MARK-1003 was already dropped in the herbarium of the biology division, Faculty of Sciences Semlalia, Marrakech, Morocco. Plant extraction was performed as described before [22]. Briefly, powder was obtained by crushing the dried roots, and was then extracted with distillated water (1 g/10 mL) under agitation for 24 h. Aqueous macerate was centrifuged (1200 rpm), filtered and lyophilized in the Christ instrument, then stored at 4 °C until its use. 

The assessment of toxicity at various doses (1000, 2000, and 5000 mg/kg) for the aqueous extract of *Anacyclus pyrethrum* confirmed its safety. Over a 14-day period of AEAP administration, no instances of mortality or significant alterations in body or organ weights (*p* > 0.05) were observed. The Lethal Dose 50 (LD50) value for AEAP exceeded 5000 mg/kg, underscoring the extract’s non-toxic nature (Supplementary File).

### 2.2. AEAP Metabolite Profiling

The chemical composition of AEAP was determined using high performance liquid chromatography (HPLC) from Thermo Electron Corporation (Waltham, MA, USA) with a Zorbax Eclipse XDB-C18 reversed-phase column (4.6 × 150 mm, 3.5 µm, Agilent, Santa Clara, CA, USA). The setup included quaternary pumps, an online degasser, and an injection valve with a 20 μL loop (Rheodyne, Rohnert Park, CA, USA). The system was coupled to a photodiode array (PDA) detector (model SPD-M20A) and a mass spectrometer featuring an ion-trap analyzer and an electrospray ionization source. Before HPLC-PDA-MS/MS analysis, the extract was dissolved in MeOH:MTBE (1:1) and filtered through 0.22 μm Millipore membranes. The mobile phase consisted of water and acetonitrile (ACN), each with 0.1% formic acid (FA). A linear gradient was applied, starting with 5% ACN and increasing to 30% over 60 min, then to 90% over the following 30 min. The flow rate was set to 1 mL/min and the column temperature maintained at 29 °C with a split ratio of 1:1. Full scan acquisition mode was used, scanning spectra from *m*/*z* 50 to 2000 in the negative mode. Data were analyzed using X-calibur software (X-calibur™ 2.0.7, Thermo Fisher Scientific, Waltham, MA, USA).

### 2.3. Animals 

Adult male Sprague-Dawley rats, aged 18–19 weeks and weighing between 180 and 230 g, were sourced from the animal facility at the Semlalia Faculty of Sciences, Cadi Ayyad University, Marrakech, Morocco. The rats were housed in an isolated chamber with controlled temperature (22 ± 2 °C) and humidity (50 ± 10%), maintained on a 12 h light/dark cycle. They were kept in transparent cages with five rats per cage and had free access to food and water. All procedures involving animals adhered to the EU2010/63 European Council Directive for the Care and Use of Laboratory Animals. The Institutional Review Board of the Faculty of Sciences at Cadi Ayyad University in Marrakech, Morocco, provided ethical approval for the experiments, complying with the Committee for the Regulation and Oversight of Animal Experimentation and Animal Ethics guidelines. This study was assigned the protocol code BAM1212/04/23, receiving approval in April 2023. After a period of acclimatization to the laboratory environment, the animals were categorized into five groups (6 animals per group): (1) control group: received vehicle (saline solution 0.9%); (2) group treated with AEAP: 200 mg/kg (orally) for 30 days; (3) Fentanyl-dependent group: 30 days of daily administration of Fentanyl followed by a withdrawal phase (7 days); (4) Fentanyl + post-AEAP: Fentanyl-dependent group after 30 days of daily administration of Fentanyl and post-treated with AEAP 200 mg/kg for 7 days to assess the potential curative effects of AEAP; and (5) Fentanyl + co-AEAP: administration of AEAP 200 mg/kg was followed by Fentanyl 30 min later for 30 days—this co-treatment phase aimed to explore the potential preventive effects of AEAP and a preliminary study was conducted before examining its impact on Fentanyl withdrawal. This initial investigation assessed the pharmacological effects of various AEAP doses (100 mg/kg, 200 mg/kg, 400 mg/kg, and 800 mg/kg) on anxiety and depression. The findings indicated that 200 mg/kg had the most notable therapeutic effect, which led to its selection for the main study.

### 2.4. Drug Administration

Fentanyl was acquired commercially, dissolved in saline solution, and administered using a progressively escalating dose based on prior studies [23]. The dosing started at 20 μg/kg/day and was increased by 20% each week, reaching a maximum of 40 μg/kg/day, following protocols from previous research [4,24,25]. The drug was administered by intraperitoneal injection, as a daily chronic administration for 30 days (from day 3 to day 33), mimicking the progressive tolerance that characterizes human addiction. 

### 2.5. Behavioral Assessment

#### 2.5.1. Conditioned Place Preference (CPP)

Fentanyl-induced CPP was carried out following the previously established protocol [22]. The CPP system comprised three PVC compartments, including two large side chambers (30 × 25 × 30 cm) with distinct floor textures serving as somatosensory cues, and one central chamber (11 × 25 × 30 cm). The CPP protocol involved three phases: pre-conditioning, conditioning, and dependence. 

During the pre-conditioning phase (days 1 to 3), each animal was placed in the central chamber with unrestricted access to the entire apparatus (doors removed), and its activity was recorded for 15 min. On day 3, the time spent and the number of entries (defined as having all four legs inside a chamber) were calculated. Animals exhibiting an initial preference for one side chamber were excluded from the study to avoid bias.

The conditioning phase (days 4 to 9): During this phase, each rat from experimental groups was treated for 6 days with alternate injections of either drug or saline, alternatively in the morning (10:00) and evening (20:00) sessions. Rats were immediately confined after each injection into their drug-paired (zebra chamber) or saline-paired (white chamber) chambers for 45 min, and doors were put on. Controls received only vehicle during alternating sessions. On day 9, animals were allowed free access to both chambers and were recorded for 15 min. Animals who had a drug aversive effect were removed after calculating the number of entries to the drug-paired chamber/total entries, which is mentioned as the CPP score, and measuring the time spent in the drug-paired chamber [26]. The dependence phase (days 10 to 33): During this phase, experimental animals received drugs daily (at 10:00 am) and were put into the drug-paired chamber for 45 min (doors closed); control groups continued vehicle injection. The withdrawal phase (day 40): Animals were allowed free access to both chambers and were recorded for 15 min.

#### 2.5.2. Open Field Test (OFT)

To evaluate exploration and locomotor activity, the Open Field Test (OFT) was used. It consists of a white arena (80 × 80 × 40 cm) divided into 25 equal squares. The animals were placed individually for 10 min, and the number of lines crossed with all four legs and rearing were recorded [27]. After each rat’s test, the OFT apparatus underwent cleaning using a 10% ethanol solution to remove any potential residual olfactory cues.

#### 2.5.3. Porsolt’s Forced Swim Test (FST)

Rats were individually subjected to the Forced Swim Test in an open cylindrical tank (21 × 60 cm) filled with 40 cm of water at a temperature of 25 ± 1 °C. Immobility was recorded during a 10 min session starting from the test’s onset. Immobility was defined as the rats remaining motionless in the water, without performing active behaviors like jumping, diving, or swimming, and only making movements necessary to keep their heads above water. Prolonged periods of immobility were indicative of a depressant-like effect in their behavioral response [28].

#### 2.5.4. Elevated Plus Maze (EPM)

To assess anxiety-like behaviors, the Elevated Plus Maze (EPM) is widely used among rodents. The apparatus is raised 100 cm from the floor and contains two open arms and two enclosed arms (each measuring 50 × 10 cm), as well as a central zone (10 × 10 cm) where the animal is first placed. During a 10 min period, the number of entries (four legs on the arm) and time invested in the closed and open arms were measured [29]. At the end of the test, the floor was cleaned with 10% ethanol.

**Figure 1:**
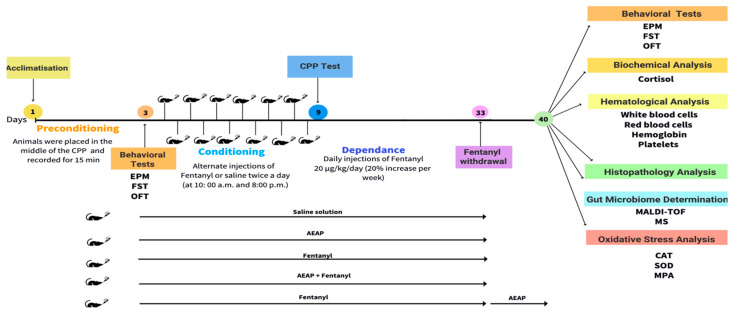
Graphical flowchart illustrating the experimental design, including the selection of rats, drug administration, behavioral tests, and sample collection for gut microbiota and oxidative stress analysis to investigate the modulation of the gut microbiome in Fentanyl-induced behavioral and biochemical impairment in rats and the potential of post-treatment with *A. pyrethrum* L. aqueous extract to mitigate adverse effects.

Behavioral assessments were performed during the preconditioning phase (day 1) and the withdrawal phase (day 40) (Figure 1).

### 2.6. Biochemical and Hematological Analyses

After the behavioral assessment during the withdrawal phase (day 40), animals were sacrificed by cervical dislocation under ether anesthesia, and a blood sample was rapidly obtained. It was collected into ice-cooled centrifugal tubes without using an anticoagulant, clotted for 30 min at 25 °C, and centrifuged for 15 min at 1500× *g* to collect the serum, which was stored at −20 °C for biochemical measurement. Cortisol levels and hematologic function (white and red blood cells and platelets) were quantified using the standard technique with a biochemical machine (Cobas 6000, Roche, Basel, Switzerland).

### 2.7. Oxidative Stress

The collected animal brains were softly removed and placed on a cold surface to maintain tissue integrity. The hippocampus, a region of interest for this study, was then dissected from each brain sample. To ensure consistency, the dissection was performed using precise anatomical landmarks, such as the surrounding structures and coordinates based on a standardized brain atlas [30]. The hippocampus tissues were homogenized in a 20 mM Tris-HCl buffer (pH 7.4) on ice to break down the tissue into smaller fragments and create a uniform sample for subsequent biochemical analysis. The buffer solution helped maintain the stability of the extracted molecules during the homogenization process and subsequent analysis.

#### 2.7.1. LPO Assay

Lipid peroxidation levels were evaluated by measuring thiobarbituric acid-reactive substances (TBARS) in the homogenates using a previously described protocol [31]. Briefly, 100 mg of crude hippocampal homogenate was centrifuged at 4 °C (1000× *g* for 10 min). The supernatant was mixed with 1 mL of 10% trichloroacetic acid (TCA) and 1 mL of 0.67% thiobarbituric acid (TBA). The mixture was then heated in a boiling water bath for 15 min. Butanol (2:1, *v*/*v*) was added to the solution, and after further centrifugation (800 g for 5 min), the absorbance at 535 nm was measured to determine TBARS levels. Results were expressed as nmol of malondialdehyde (MDA) per gram of wet tissue.

#### 2.7.2. CAT Activity

Catalase (CAT) activity was assessed using the hydrogen peroxide (H_2_O_2_)-dependent method to measure the production of water (H_2_O) and oxygen (O_2_) [32]. In brief, 0.05 mL of the sample was combined with 1 mL of 0.019 M H_2_O_2_ and 1.95 mL of 50 mM phosphate buffer in a 3 mL quartz cuvette. The absorbance at 240 nm was recorded at the start (T_0_) and then every 30 s for 2 min. CAT activity was calculated as mmol of H_2_O_2_ decomposed per minute per milligram of protein.

#### 2.7.3. SOD Activity

Superoxide dismutase (SOD) activity was measured by its capacity to inhibit the photoreduction of nitro blue tetrazolium (NBT) using the spectrophotometric method [33]. The assay mixture included 2.4 × 10^−6^ M riboflavin, 0.01 M methionine, 1.67 × 10^−4^ M NBT, and 0.05 M potassium phosphate buffer (pH 7.4) at 25 °C, with the absorbance recorded at 560 nm. One unit of SOD activity was defined as the amount of enzyme required to achieve a 50% reduction in the NBT reduction rate.

### 2.8. Gut Microbiota Determination

Following the behavioral tests, 1 cm intestinal samples were collected at euthanasia from each group to assess the effects of Fentanyl and AEAP on bacterial density and relative abundance. Intestinal tracts were promptly placed into sterile tubes. Each sample was then uniformly spread on the surface of sterile, dry nutrient agar medium in Petri dishes. The samples were incubated at 37 °C for 72 h under both aerobic and anaerobic conditions [22]. After incubation, bacterial colonies were carefully counted, and their identification was performed using Matrix-Assisted Laser Desorption Ionization–Time of Flight Mass Spectrometry (MALDI-TOF MS).

#### 2.8.1. MALDI-TOF MS

Following the methodology outlined previously [34], bacterial identification was performed using the cell smear (direct smear) approach. This identification method involves direct transfer of a single colony on a polished steel MSP 96 target (Bruker Daltonics, Billerica, MA, USA). Before covering the bacterial spot with matrix α-cyano-4-hydroxycinnamic acid (CHCA) (Sigma-Aldrich, St. Louis, MO, USA) solution, 1 μL of 70% formic acid solution was applied to increase the probability of accurate identification. When the matrix has been added and dried, the plate is inserted into the MS for analysis.

#### 2.8.2. MALDI-TOF MS Analysis

The Bruker Daltonics Microflex LT mass spectrometer captured and interpreted mass spectra. This instrument was utilized using the research-use-only (RUO) MALDI Biotyper software (version 3.0) and the reference database V.3.1.2.0 (3995 entries). The manufacturer’s bacterial test standard and instructions were used for calibration (Bruker Daltonics). Each strain was evaluated twice from different preparations.

MALDI-TOF MS manufacturer criteria were used to analyze the results. Species scored 2.0, whereas genera scored 1.7 to 2.0. Scores below 1.7 were not assigned identities.

### 2.9. Histological Study

After completing the previous tests, the intestines were dissected and submerged in a 10% formalin solution overnight. The organs then underwent dehydration through a series of graded alcohols before being embedded in paraffin wax. Sections, 4 μm thick, were prepared and stained with hematoxylin and eosin for pathological analysis, following the protocol described by Malatesta (2016) [35].

### 2.10. Statistical Analyses

The data were analyzed and presented as mean ± standard error of the mean (SEM) using GraphPad Prism 9 (San Diego, CA, USA). The results, expressed as mean ± SEM (n = 6), underwent distinct statistical analyses based on the nature of the assessments. For the evaluation of behavioral assessments at both initial and withdrawal phases, a two-way analysis of variance (ANOVA) was employed, considering the influence of two variables: different treatment groups and the timing factor (initial and withdrawal). However, biochemical, oxidative stress, and gut microbiota analyses were conducted solely during the withdrawal phase, necessitating the utilization of a one-way ANOVA. Post-hoc comparisons were conducted using the Tukey test when *p* < 0.05. A *p*-value less than 0.05 was considered statistically significant.

## 3. Results

### 3.1. HPLC–PDA–MS Aqueous Extract of Anacyclus Pyrethrum Profiles

**Figure 2:**
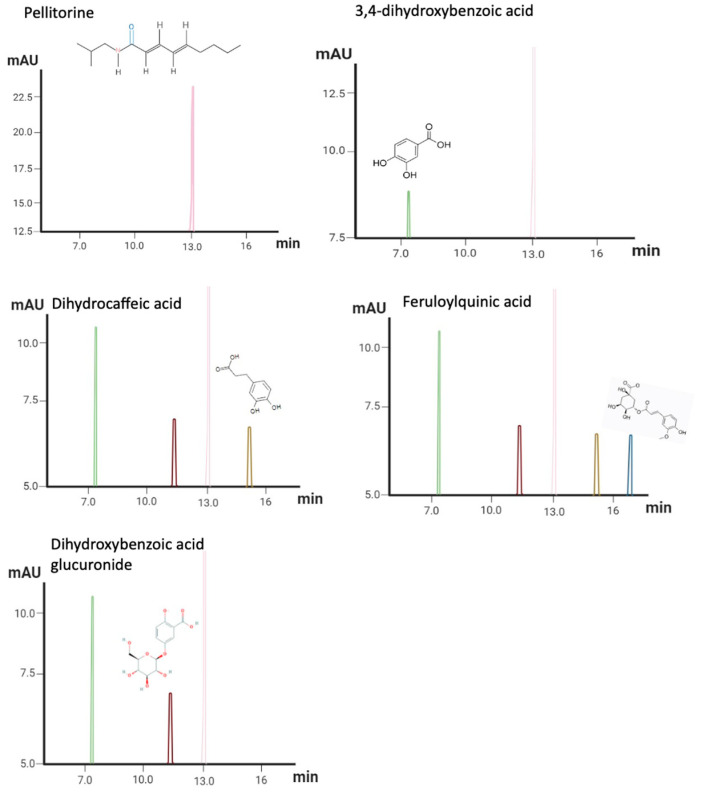
Chromatograms and structural representation of the predominant secondary metabolites found in AEAP. HPLC–PDA–MS/MS chromatograms of compounds isolated from AEAP, peak identification, and characterization are given in Figure S1 and Table S1.

HPLC–PDA–MS profiling of AEAP produces extremely rich metabolite profiles (Table S1 and Figure S1). The top five most frequent metabolites in AEAP represented a diverse range of chemical classes: alkamide (pellitorine; C_14_H_25_NO; MW = 223 g/mol), benzoic acid derivative (3,4-Dihydroxybenzoic Acid, also referred to as Protocatechuic Acid; C_7_H_6_O_4_; MW = 153 g/mol), glucuronide of dihydroxybenzoic acid (Dihydroxybenzoic Acid Glucuronide; C_13_H_12_O_9_; MW = 329 g/mol), phenolic acid (Dihydrocaffeic Acid; C_9_H_10_O_4_; MW = 107 and 135), and phenolic acid derivative, specifically a derivative of cinnamic acid (Feruloylquinic Acid; C_16_H_18_O_9_; MW = 367 g/mol) (Figure 2).

### 3.2. Fentanyl-Induced Conditioned Place Preference (CPP)

**Figure 3:**
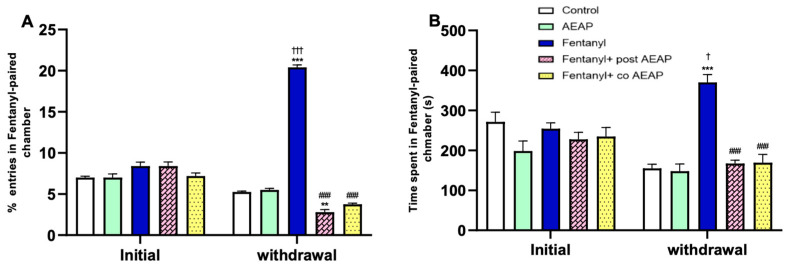
Fentanyl and/or aqueous extract of *Anacyclus pyrethrum* (AEAP) effects on dependence of conditioned place preference (CPP); (**A**) % of entries and (**B**) time spent in Fentanyl-paired chamber during the CPP test. Data represent mean ± SEM (n = 6 per group). Data were analyzed by two-way ANOVA with post-hoc Tukey HSD (Honestly Significant Difference) (phase × group), and one-way ANOVA during withdrawal. * vs. vehicle during withdrawal; ^†^ vs. initial; ^#^ vs. Fentanyl during withdrawal. ^†^ *p* < 0.05; ** *p* < 0.01; ^†††^ or ^###^ or *** *p* < 0.001.

Figure 3 presents the findings from the CPP test, which evaluates the rewarding properties of psychoactive substances. The two-way ANOVA analysis, examining the effects of time and group, revealed a significant difference in the percentage of entries (F_(4,50)_ = 216.4; *p* < 0.05) and time spent (F_(4,50)_ = 10.94; *p* < 0.05) in the Fentanyl-paired chamber (Figure 3A,B). During the withdrawal phase, the group withdrawn from Fentanyl showed a significant increase in entries and time spent in the drug-paired chamber compared to the initial phase and the control group during withdrawal (*p* < 0.05) (Figure 3A,B). The one-way ANOVA test revealed that concurrent administration of AEAP at a daily dose of 200 mg/kg significantly reduced the number of entries (F_(4,25)_ = 216; *p* < 0.05) and time spent in the conditioned chamber (F_(4,25)_ = 33.74; *p* < 0.05) during the withdrawal phase. Additionally, the results showed that administering AEAP at the same dosage for one week during the withdrawal period resulted in a significant decrease in the number of entries and time spent in the paired chamber (*p* < 0.05).

### 3.3. Fentanyl Withdrawal-Induced Anxiety and Depression and the Potential of AEAP to Alleviate Adverse Outcomes

**Figure 4:**
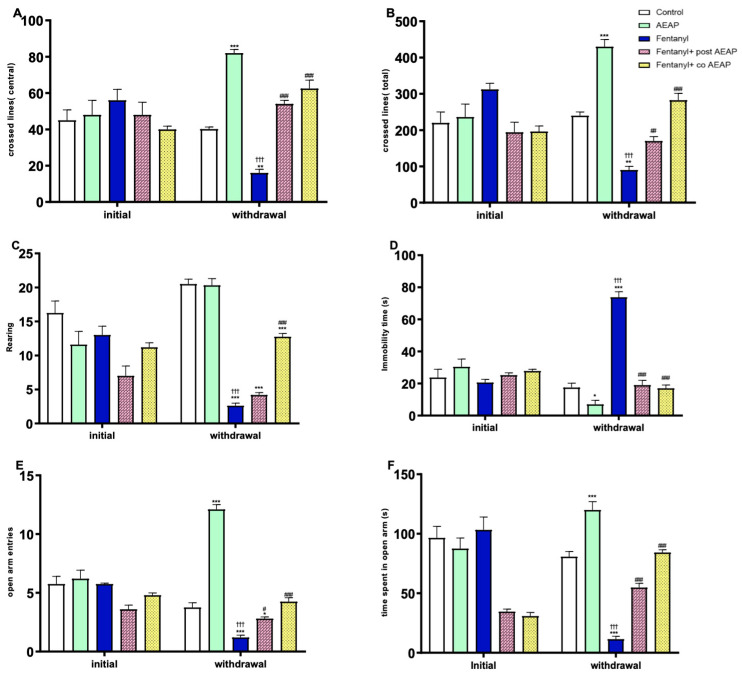
Fentanyl and/or aqueous extract of *A. pyrethrum* (AEAP) effects on (**A**) the central crossed lines, (**B**) the total crossed lines, (**C**) the number of rearings in the OFT, (**D**) the immobility time in FST, (**E**) the open arm entries, and (**F**) the time spent in the open arms in the EPM. Data represent mean ± SEM (n = 6 per group). Data were analyzed by two-way ANOVA with post-hoc Tukey HSD (Honestly Significant Difference) (phase × group), and one-way ANOVA during withdrawal. * vs. vehicle during withdrawal; ^†^ vs. initial; ^#^ vs. Fentanyl during withdrawal. * or ^#^ *p* < 0.05; ** or ^##^ *p* < 0.01; ^†††^ or ^###^ or *** *p* < 0.001.

The two-way ANOVA revealed significant differences between phases (initial and withdrawal) and groups for various behavioral parameters. Specifically, in the OFT, significant effects were observed in central crossed lines (F_(4,50)_ = 18.59; *p* < 0.05), total crossed lines (F_(4,50)_ = 25.71; *p* < 0.05), and rearings (F_(4,50)_ = 20.30; *p* < 0.05). Additionally, in the FST, significant differences were found in immobility (F_(4,50)_ = 46.58; *p* < 0.05). Furthermore, in the EPM, significant effects were noted in time spent (F_(4,50)_ = 40.85; *p* < 0.05) and entries in open arms (F_(4,50)_ = 46.14; *p* < 0.05) (Figure 4A–E).

Subsequent to withdrawal, our observations using the OFT demonstrated that the administration of a daily dose of 200 mg/kg of AEAP alone resulted in a highly significant increase in the number of central and total lines crossed (F_(4,25)_ = 88.38, *p* < 0.05 and F_(4,25)_ = 73.84, *p* < 0.05, respectively) (Figure 4A,B). Additionally, AEAP administration led to a prolonged duration spent in the open arms of the EPM (F_(4,25)_ = 88.50; *p* < 0.05) (Figure 4E,F). Moreover, AEAP induced a decrease in the immobility time during the FST compared to control during withdrawal (F_(4,25)_ = 91.24; *p* < 0.05).

Following withdrawal, the administration of Fentanyl resulted in a significant decrease in central and total lines crossed, as well as rearing behavior, as observed in the OFT (Figure 4A–C). Additionally, it caused a highly significant reduction in the time spent and the number of entries into the open arms of the EPM (*p* < 0.05) (Figure 4E,F). Furthermore, Fentanyl elicited a significant increase in immobility time during the FST (*p* < 0.05) (Figure 4D), suggesting that Fentanyl induces a decrease in locomotor activity and promotes depressive and anxious-like behaviors during withdrawal. In contrast, AEAP treatment mitigated these behaviors. In the group withdrawn from Fentanyl and subsequently treated with AEAP, anxiety and depressive-like behaviors significantly decreased compared to the initial phase (*p* < 0.01) and to the control during withdrawal. Furthermore, in the co-treatment group receiving both AEAP and Fentanyl, the behavioral withdrawal syndrome characterized by anxiety and depressive behaviors was not observed (Figure 4D,F).

### 3.4. Fentanyl Withdrawal-Induced Cortisol Level Elevation

**Figure 5:**
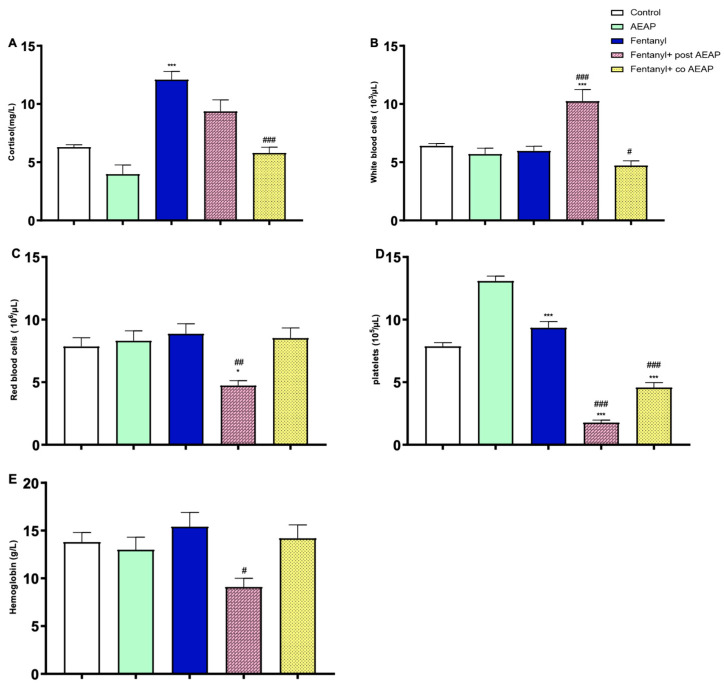
Fentanyl and/or aqueous extract of *A. pyrethrum* (AEAP) effects on (**A**) cortisol, (**B**) white blood cells, (**C**) red blood cells, (**D**) platelets, and (**E**) hemoglobin during the withdrawal phase. Data represent mean ± SEM (n = 6 per group). Data were analyzed by one-way ANOVA with post-hoc Tukey HSD (Honestly Significant Difference). * vs. control; ^#^ vs. Fentanyl. * or ^#^ *p* < 0.05; ^##^ *p* < 0.01; *** or ^###^ or *p* < 0.001.

During withdrawal, the biochemical and hematological profiles of the groups exhibited significant differences, as determined by the one-way ANOVA. Specifically, significant variations were observed in cortisol levels (F_(4,25)_ = 21.36; *p* < 0.05), white blood cell counts (F_(4,25)_ = 14.00; *p* < 0.05), red blood cell counts (F_(4,25)_ = 5.48; *p* < 0.05), platelet counts (F_(4,25)_ = 135.7; *p* < 0.05), and hemoglobin levels (F_(4,25)_ = 3.72; *p* < 0.05) (Figure 5).

In Figure 5A, it can be observed that cortisol levels increased during Fentanyl withdrawal when compared to the control group (*p* < 0.05). However, in the group withdrawn from Fentanyl and subsequently treated with AEAP, cortisol levels significantly decreased compared to the aforementioned group (*p* < 0.05) (Figure 5A). Additionally, there was a significant decrease in cortisol levels for the treated group receiving combined AEAP and Fentanyl, indicating the potential of AEAP to alleviate stress. Additionally, neither chronic administration of Fentanyl nor AEAP had a significant effect on white blood cells, red blood cells, or hemoglobin levels (Figure 5B–E). Conversely the post-treatment with AEAP induced a significant increase in white blood cells (*p* < 0.05), and a significant decrease in hemoglobin (*p* < 0.05), red blood cells (*p* < 0.05), and platelets levels (*p* < 0.05) (Figure 5B–E).

### 3.5. Fentanyl Withdrawal-Induced Oxidative Stress and the Antioxidant Capacity of AEAP

**Figure 6:**
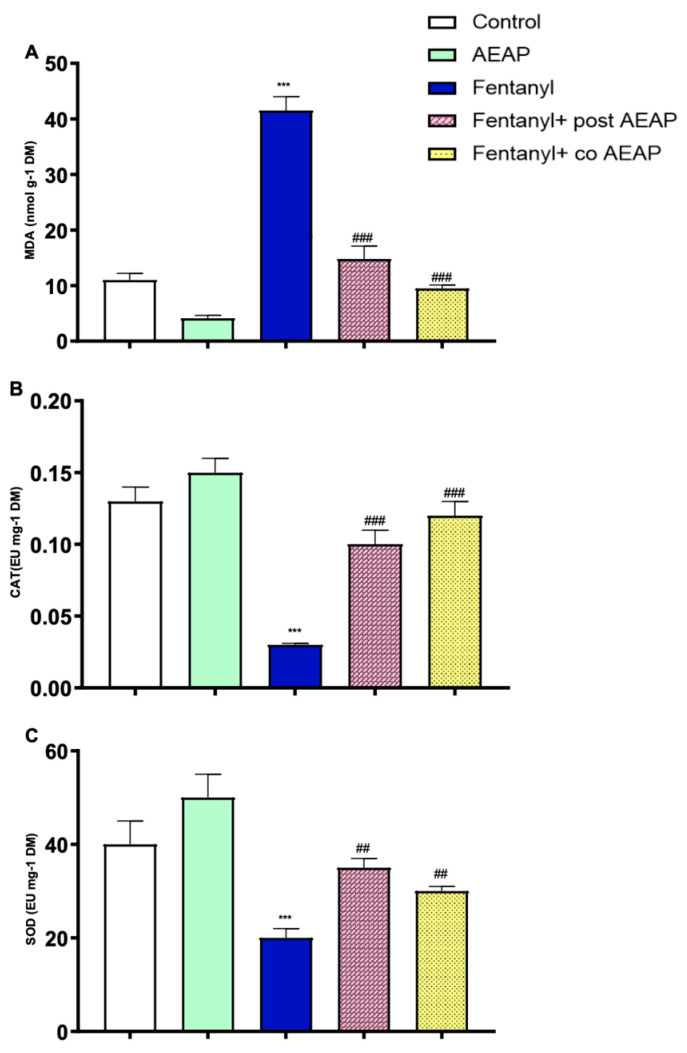
Fentanyl and/or aqueous extract of *A. pyrethrum* (AEAP) effects on oxidative stress: (**A**) MDA, (**B**) CAT, and (**C**) SOD during the withdrawal phase. Data represent mean ± SEM (n = 6 per group). Data were analyzed by one-way ANOVA with post-hoc Tukey HSD (Honestly Significant Difference). * vs. control; ^#^ vs. Fentanyl. ^##^ *p* < 0.01; *** or ^###^ *p* < 0.001.

The evaluation of oxidative stress markers, including MDA, CAT, and SOD, offers valuable insights into cellular redox balance and damage. In this study, a one-way ANOVA revealed significant differences between groups during withdrawal, as evidenced by MDA (F_(4,25)_ = 79.65; *p* < 0.05), CAT (F_(4,25)_ = 26.56; *p* < 0.05), and SOD (F_(4,25)_ = 10.59; *p* < 0.05) levels. A notable increase in MDA levels was observed in the Fentanyl-withdrawn group (41.50 nmol g^−1^) compared to the control group (11.01 nmol g^−1^, *p* < 0.05). Moreover, CAT and SOD antioxidant activities were significantly reduced in the Fentanyl-withdrawn group (*p* < 0.05 compared to control). However, post-treatment with AEAP resulted in a significant reduction of MDA levels, indicating its protective effects against the damage caused by chronic Fentanyl administration. Additionally, following co- and post-treatment with AEAP, SOD and CAT levels increased in all groups, suggesting the positive effects of the treatment on redox balance and cellular protection (Figure 6A–C).

### 3.6. Alterations in Microbiota Density and Composition during Fentanyl Withdrawal and the Ameliorative Effect of AEAP

**Figure 7:**
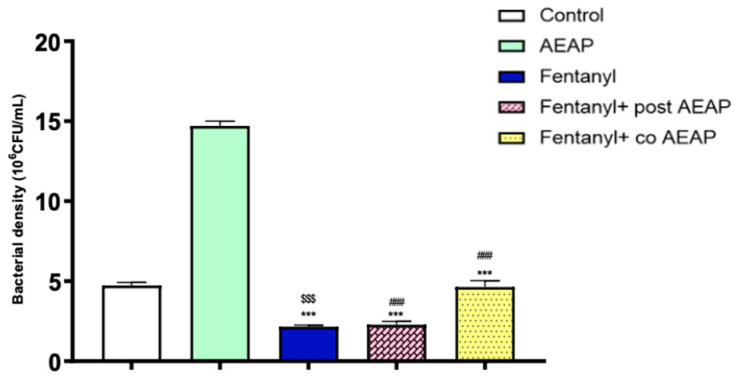
Fentanyl and/or aqueous extract of *A. pyrethrum* (AEAP) effects on bacterial density in gut microbiota during the withdrawal phase. The density represents the total effective bacteria in the sample. Data represent mean ± SEM (n = 6 per group). Data were analyzed by one-way ANOVA with post-hoc Tukey HSD (Honestly Significant Difference). * vs. control; ^#^ vs. Fentanyl; ^$^ vs. AEAP. *** or ^###^ or ^$$$^ *p* < 0.001.

Figure 7 illustrates significant variations in microbial abundance across different samples during withdrawal (F_(4,25)_ = 394.8; *p* < 0.05). AEAP alone exhibited the highest bacterial density; in contrast, the Fentanyl-withdrawn group showed significantly lower bacterial density compared to the control group (*p* < 0.5), suggesting the detrimental influence of chronic administration of opioids on GM. However, the damage caused by this substance was partially reversible, as the co- and post-treatment with AEAP significantly increased the density of bacteria compared to the Fentanyl-withdrawn group.

**Figure 8:**
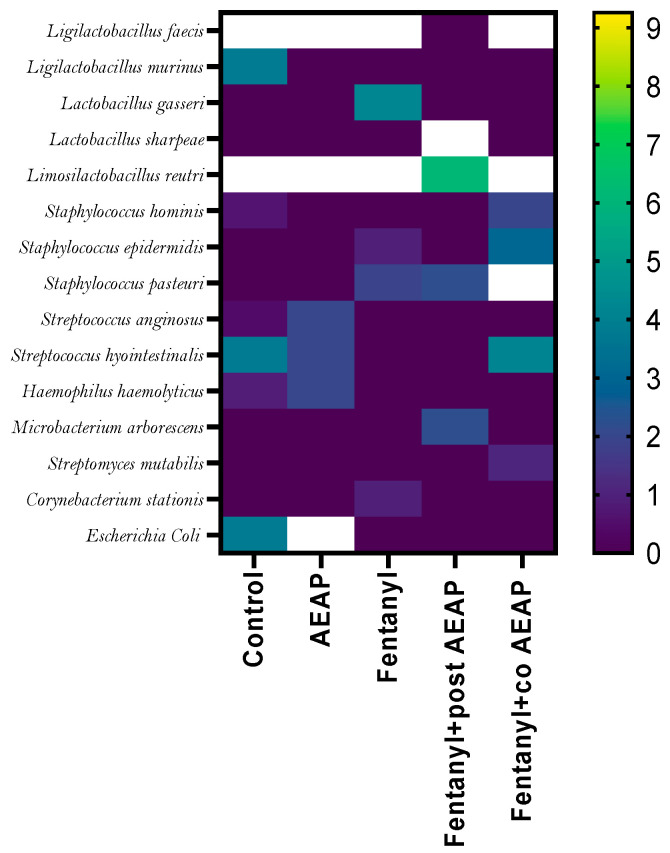
Community heatmap showing microbiota abundance at species level as a percentage of total bacterial sequences. The columns correspond to the groups. The rows correspond to the microbiota in species level.

Figure 8 presents a list of identified bacterial species along with their relative abundances and frequencies. GM is composed of multiple bacterial species, each with varying relative abundances. AEAP administration increased the diversity of GM by enhancing the levels of bacteria such as *Ligilactobacillus*, *Escherichia coli*, and *Staphylococcus*. On the other hand, it decreased the abundance of *Rodentibacter* and *Corynebacterium*. The Fentanyl-withdrawn group exhibited dysbiosis in the bacterial abundance profile, with significantly decreased levels of *Staphylococcus* and *Escherichia coli* compared to the other groups. However, in the co- and post-AEAP groups, the abundance of *Escherichia coli* increased compared to the Fentanyl-treated group, indicating the potential restorative effects of AEAP on the GM composition (Figure 8 and Supplementary Table S2).

### 3.7. Alterations in Intestinal Tissue during Fentanyl Withdrawal and the Ameliorative Effect of AEAP

**Figure 9:**
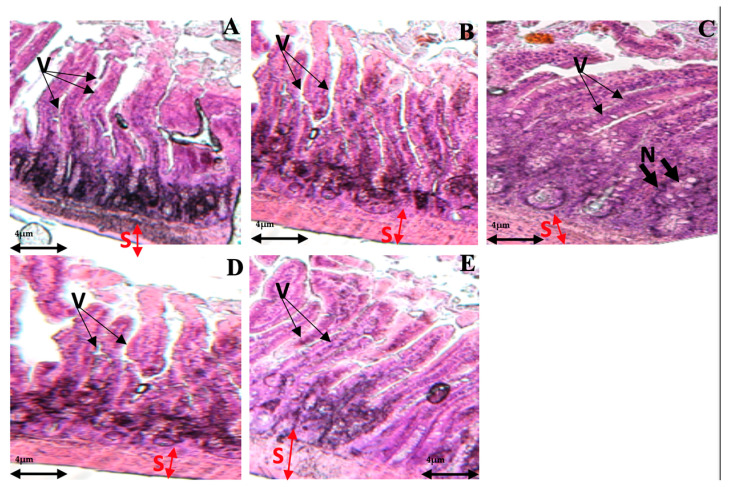
Effects of Fentanyl and/or aqueous extract of *A. pyrethrum* (AEAP) on the histopathological tissues in the small intestines. Samples were examined using an optical microscope (×200) for the following groups: (**A**) control group, (**B**) AEAP group, (**C**) Fentanyl-withdrawn group, (**D**) Fentanyl + post-AEAP group, and (**E**) Fentanyl + co-AEAP. Notable histopathological changes were identified, including alterations in villi structure (V), submucosal layer (S), and the presence of large fissures indicative of villus necrosis (N). Scale bar = 4 μm.

In Figure 9, distinct histological observations were made across the different treatment groups. The control group and animals treated solely with AEAP exhibited regular villi and gland structures without any signs of inflammatory cell infiltration (Figure 9A), indicative of a healthy mucosal architecture. However, in the groups treated with Fentanyl, there was a notable presence of severe mucosal edema accompanied by infiltration of necrotic epithelial and inflammatory cells. This demonstrated the substantial impact of Fentanyl on intestinal morphology. In contrast, the group co-treated with AEAP prior to Fentanyl administration (Fentanyl + co-AEAP) showed a significant absence of substantial edema or necrotic mucosal villi, suggesting the protective efficacy of AEAP against Fentanyl-induced damage. On the other hand, the groups receiving AEAP post-Fentanyl exposure (curative groups) exhibited a milder edema of intestinal villi, with partial destruction observed in some villi. This finding suggests a moderate protective effect of AEAP when administered after Fentanyl exposure compared to its pre-emptive use. These distinct histological findings highlight the protective effects of AEAP, showcasing its potential in mitigating Fentanyl-induced damage when administered both before and after Fentanyl exposure. The comparison among treatment groups underscores the varying degrees of mucosal alterations and emphasizes the beneficial impact of AEAP in preserving intestinal histology amidst Fentanyl-induced damage.

## 4. Discussion

The aim of this study was to examine for the first time the relationship between GM and oxidative stress during OUD and to explore the therapeutic potential of *A. pyrethrum* in mitigating the associated adverse effects during withdrawal.

Following the cessation of chronic administration of Fentanyl, notable alterations in behavior were observed, characterized by an increased duration and frequency of visits to the Fentanyl compartment in the CPP test, suggestive of preference inclinations. Simultaneously, signs of anxiety and depressive-like behaviors emerged, alongside a reduction in locomotor activity. Analysis of blood samples revealed heightened cortisol levels, indicating an elevated stress response during withdrawal. Additionally, a decrease in both the density and diversity of the gut microbiota was observed, accompanied by evidence of increased oxidative stress manifested by elevated levels of malondialdehyde (MDA) and decreased levels of catalase (CAT) and superoxide dismutase (SOD) [1].

Results of the CPP test showed that a daily dose of Fentanyl increased the number of entries to the paired chamber and the time spent in the conditioned chamber during withdrawal. The drug-paired chamber is expected to produce feelings of pleasure as a conditioned response, leading to approach behaviors toward, and increased time spent in, the drug-paired chamber [36]. The rewarding properties are linked mainly to dopamine release and dopaminergic projections to the reward system [37]. The Fentanyl-dependent groups exhibited a decrease in locomotor activity, as evidenced by a reduced number of central crossed lines in the OFT. Additionally, these groups showed an increase in the number of transitions and time spent in the closed arm of the EPM, indicating anxious-like behavior. Furthermore, the dependent groups displayed a depressive-like behavior, characterized by an increased immobility time in the FST. These findings align with previous studies that have consistently reported the presence of depressive- and anxious-like behaviors as common withdrawal symptoms associated with OUD [38,39,40,41].

The manifestation of these symptoms is often linked to a decrease in the release of neurotransmitters such as dopamine, oxytocin, and serotonin following the discontinuation of drug use. During the early stages of addiction, the consumption of drugs is primarily motivated by the immediate rewarding effects, also known as the “positive” effects, which are mediated by the mesocorticolimbic dopamine systems [42,43,44]. During this phase, the initial positive reinforcement associated with immediate drug consumption gradually transforms into negative reinforcement. In this context, individuals use the drug to avoid or alleviate negative emotional states such as anxiety, irritability, and dysphoria that arise when access to the drug is restricted. The drug serves as a means to prevent the onset of these negative emotional states [44]. The transition toward behavior driven by negative reinforcement, specifically withdrawal, is primarily mediated by brain stress systems. These systems include corticotropin-releasing factor (CRF), which is active within the hypothalamic–pituitary–adrenal (HPA) axis, as well as the extended amygdala. These neural pathways play a significant role in the manifestation of withdrawal symptoms and the associated negative emotional states [45].

The Fentanyl-dependent group also exhibited a heightened oxidative stress, as evidenced by increased levels of MDA and decreased concentrations of CAT and SOD. Consistent with our findings, previous studies have substantiated that chronic opioid exposure induces oxidative stress through diverse mechanisms, including μ-opioid receptors, phospholipase D2, and NADPH oxidase [46]. Opioids possess the capacity to activate opioid receptors, initiating the production of free radicals such as reactive oxygen (ROS) or reactive nitrogen (RNS) species, and concurrently diminishing antioxidant activities within target cells [47]. This direct activation of opioid receptors has been implicated in the generation of oxidative stress associated with opioid use disorder, thereby contributing to the development of opiate dependence [48]. Fentanyl could induce oxidative stress through an indirect pattern also, due to its capacity to modulate GM. Our findings revealed a significant decrease in both the density and diversity of GM during OUD. GM generates various metabolites, such as short-chain fatty acids, that can impact the body’s redox status and biological processes [49]. It was also proved that GM can influence the vulnerability of the brain to oxidative stress, which has been suggested as a contributing factor to several disorders [50]. It is worth noting that alterations in GM have been associated with various conditions, including depression, anxiety disorders, autism spectrum disorders, and neurodegenerative diseases [51,52,53]. The GM can influence the brain through multiple pathways, including the gut–brain axis, modulation of the immune system, and production of microbial metabolites that can cross the blood–brain barrier. The GM has the potential to directly influence the immune system through the activation of the vagus nerve [54,55,56,57], thereby initiating bidirectional communication with the CNS [58]. Furthermore, the GM’s indirect impact on the innate immune system can lead to changes in the levels of circulating pro- and anti-inflammatory cytokines, which directly impact brain function [52].

However, when the AEAP was co-administered alongside Fentanyl as a preventive measure, a significant amelioration of the aforementioned detrimental effects was observed. Moreover, when AEAP was post-administered during the withdrawal period, improvements in behavioral tests and cortisol levels were noted, alongside a restoration of several GM species, underscoring the curative potential of the AEAP extract in addressing OUD-induced effects. AEAP demonstrated both preventive and curative effects; however, the preventive effect remains more pronounced. The HPLC assessment has effectively detected a range of bioactive compounds in the *A. pyrethrum* extract. Phenolic compounds are known to contribute directly to antioxidative effects, while flavonoids demonstrate effectiveness in scavenging various reactive oxygen species (ROS). The presence of these secondary metabolites, along with organic acids, also contributes to the overall bioactivity of the studied plant [21]. *A. pyrethrum* is rich in polyphenols, encompassing hydroxybenzoic acid quinyl ester, caffeic acid, p-coumaric acid, and the alkylamide pellitorine. These compounds exhibit diverse beneficial effects, including the modulation of the immune system, prevention of blood clot formation, reduction of inflammation, pain relief, anticancer activity, regulation of blood sugar levels, and combatting protozoal infections [59,60].

Our study aimed to investigate the potential therapeutic application of AEAP in the treatment of OUD. AEAP contains compounds that activate opioidergic receptors, which play a role in the addictive properties of OUD. The specific compound responsible for the activation of opioid receptors in *A. pyrethrum* is not yet identified. However, some studies suggest that the plant’s extracts contain alkaloids that could be partially involved in its effects on opioid receptors and responsible for its antinociceptive properties [61,62] We observed that AEAP effectively reduced CPP parameters, commonly used to assess the rewarding properties of addictive substances. In the Fentanyl-withdrawn group post-treated with AEAP, a decrease in the number of entries and time spent in the conditioned chamber was observed, as well as significant antidepressant and anxiolytic effects. Interestingly, when AEAP was co-administered with Fentanyl, it further enhanced these beneficial behaviors compared to the opioid-dependent group. Furthermore, AEAP administration demonstrated the ability to reduce cortisol levels, indicating its potential to alleviate stress-induced withdrawal.

The findings from our study suggest a potential role for AEAP in mitigating addictive behaviors associated with OUD, which is consistent with previous research on MDMA and Trihexyphenidyl addiction [1,22]. AEAP demonstrated significant improvements in behavioral impairments associated with opioid dependence, indicating its promise as a targeted intervention for OUD. While the precise mechanism of action of AEAP remains to be fully elucidated, it is hypothesized to modulate receptors implicated in addictive effects, such as GABA, adrenergic, and dopamine receptors. These receptor interactions may contribute to the observed effects of AEAP in addressing addiction. Additionally, our previous study reported a notable reduction in cortisol levels in rats treated with AEAP compared to the control group [22], further supporting its potential therapeutic utility. The present study elucidates the potential role of AEAP in mitigating addictive behaviors associated with opioid use disorder (OUD), which is consistent with prior research findings on addiction to MDMA and Trihexyphenidyl [1,22]. AEAP exhibited significant improvements in behavioral impairments linked to opioid dependence, suggesting its promise as a targeted intervention for OUD. Although the precise mechanism of action of AEAP remains unclear, some studies have indicated its antinociceptive potential. Moreover, it has been demonstrated, through the use of opioid antagonists, that the antinociceptive effect of AEAP is partially mediated by opioid receptors [1]. Additionally, research has shown that the primary phytoconstituents present in AEAP roots are alkylamides [63], which are responsible for modulating opioidergic, serotoninergic, and GABAergic systems [61].The interaction between the opioid and GABAergic systems is crucial for understanding the rewarding and anxiolytic effects of opioids, as well as the processes involved in the development of opioid tolerance, dependence, and withdrawal [64]. We hypothesize that AEAP acts as a partial antagonist for opioids and GABAergic receptors, which could potentially explain the observed effects.

Additionally, our previous study reported a notable reduction in cortisol levels in rats treated with AEAP compared to the control group [22]. Clinical implications of our findings include the potential development of AEAP-based interventions for the treatment of OUD, which could offer a novel and effective approach to managing opioid addiction. Further research is needed to explore the specific mechanisms of action of AEAP and its potential applications in clinical settings

The application of AEAP showed an elevated proportion of *Lactobacillus*, *Rodentibacter*, *Staphylococcus*, and *Corynebacterium* compared with the addicted group. This could potentially enhance gut microbiota (GM) health by reducing endotoxin production, increasing the conversion of primary bile acids into secondary bile acids, influencing gastric tone and motility through the nigro–vagal pathway, maintaining gut immune homeostasis, and facilitating absorption [14]. Moreover, the GM has been demonstrated to play a critical role in regulating dopaminergic signaling, with over 50% of dopamine synthesis occurring in the gut [65]; this highlights the significant contribution of the GM to dopamine production and regulation. *Lactobacillus* and *Bifidobacterium*, which belong to the microbial genera Firmicutes and Actinobacteria, respectively, are commonly used as probiotics. Numerous studies have demonstrated the beneficial effects of Lactobacillus administration in reducing depression- and anxiety-like behaviors in mice [66]. By modulating the GM, *Lactobacillus* can influence dopamine availability and signaling, thereby contributing to its positive effects on mood and behavior. Oral administration of Bifidobacterium has been shown to reduce an exaggerated HPA stress response [67].

Recent studies [68,69] indicate that the GM can influence the production, metabolism, and availability of dopamine in the body, suggesting a potential link between gut microbiota, dopamine regulation, and oxidative stress. As a result, there appears to be a potential link between the GM’s influence on dopamine and its role in oxidative stress. Previous scientific papers have shown that OUD exhibits oxidative properties, which is in line with our findings. OUD has been associated with the induction of oxidative stress [70], as it impairs the antioxidant defense system by reducing the levels of various enzymatic and nonenzymatic antioxidants. Additionally, AEAP demonstrated antioxidant potential, which is consistent with other studies suggesting that this effect is attributed to the plant’s abundance of phytochemical constituents such as phenols, flavonoids, alkaloids, and tannins [71,72]. AEAP significantly mitigated the oxidative stress during OUD, indicating its beneficial effects in counteracting the oxidative damage caused by this substance.

The administration of Fentanyl resulted in behavioral impairments, altered cortisol levels, and induced oxidative stress. However, the administration of AEAP effectively mitigated these damages. Our findings also suggest that Fentanyl administration leads to GM dysbiosis, highlighting the need for further research to better understand the role of gut microbiota in substance abuse mechanisms. These findings open up new possibilities for the development of novel approaches for the treatment of OUD. It is worth noting that pellitorine, a major component of AEAP, has been reported to exhibit potential activity [61,73]. However, whether it acts alone or exhibits synergistic effects with other compounds within AEAP requires further investigation. Nevertheless, it is crucial to acknowledge the limitations of our study as it did not extensively investigate other potential mechanisms or pathways involved in addiction and its treatment. Further research is needed to delve deeper into these areas and gain a more comprehensive understanding of addiction and potential therapeutic strategies.

## 5. Conclusions

These compelling findings underscore the detrimental effects of chronic Fentanyl use, including behavioral impairments, anxiety, depressive-like behaviors, elevated stress responses, and dysregulation of cortisol levels, alongside reduced gut microbiome diversity and increased oxidative stress. However, the administration of AEAP demonstrated remarkable therapeutic potential. AEAP effectively mitigated the adverse effects of Fentanyl, leading to improved behavioral outcomes, normalized cortisol levels, and the restoration of gut microbiome species diversity. AEAP showed promise in targeting the same receptors implicated in the addictive effects of Fentanyl, suggesting its potential in addressing the underlying mechanisms of opioid dependence. AEAP exhibited antioxidant properties, countering the oxidative damage induced by chronic Fentanyl use.

The findings highlight the promise of AEAP as an innovative intervention for managing opioid use disorders, offering a novel approach to addressing the complexities of substance dependence. The potential of AEAP as a therapeutic agent opens new possibilities in the treatment landscape, emphasizing the significance of further exploration and clinical translation. This research sets the stage for future investigations into AEAP’s mechanisms of action and its application in clinical settings, paving the way for transformative advancements in the management of opioid use disorders.

## Data Availability

All data are contained in the article.

## Supplementary Materials

The following supporting information can be downloaded at: https://www.mdpi.com/article/10.3390/biomedicines12061152/s1, Figure S1: HPLC MS/MS profile of *Anacyclus pyrethrum* aqueous extract (AEAP); Table S1: Tentatively identified compounds in *Anacyclus pyrethrum* extract using HPLC-ESI-MS/MS anal; Table S2: Effects of Fentanyl and/or aqueous extract of *Anacyclus pyrethrum* (AEAP) on the abundance of the microbiota during the withdrawal phase.
