# Supplementary material for: Gut Microbiome-Mediated Mechanisms in Alleviating Opioid Addiction with Aqueous Extract of Anacyclus pyrethrum
Source: Biomedicines. 2024 May 23;12(6):1152. doi: 10.3390/biomedicines12061152 (PMC11200529; doi:10.3390/biomedicines12061152)
Supplement: Supplementary file 1 [file biomedicines-12-01152-s001.zip › biomedicines-2985258-supplementary.pdf]

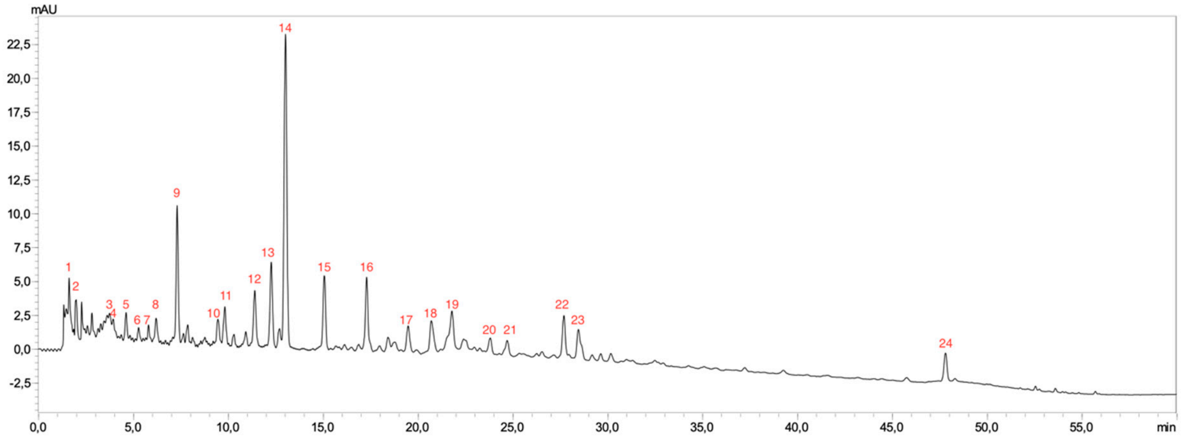

Figure S1. HPLC MS/MS profile of *A. pyrethrum* aqueous extract (AEAP)

(1) Citric acid
(2) Gallic acid derivative
(3) Dihydroxybenzoic acid derivative
(4) Dihydroxybenzoic acid rhamnoside
(5) Dihydroxybenzoic acid glucoside
(6) Caffeoylglucaric acid
(7) Gallic acid
(8) Vanillyl quinic acid
(9) 3,4-dihydroxybenzoic acid
(10) Hydroxybenzoic acid glycerol
(11) Chlorogenic acid
(12) Hydroxybenzoic acid quinyl ester
(13) Dihydroxybenzoic acid glucuronide
(14) Pellitorine
(15) Dihydrocaffeic acid
(16) Feruloylquinic acid
(17) Cryptochlorogenic acid
(18) Caffeic acid
(19) p-Coumaric acid
(20) p-Coumaroylquinic acid
(21) Hydroxycoumarin
(22) Isochlorogenic acid C
(23) Quercetin rhamnoside
(24) Isochlorogenic acid C

Table S1. Tentatively identified compounds in *A. pyrethrum* extract using HPLC-ESI-MS/MS analysis.

Peak #	Rt (min)	$[M.W]^-$	MS/MS	Compound name
1	1.516	191	111	Citric acid
2	1.935	335	169	Gallic acid derivative
3	3.523	267	108, 153	Dihydroxybenzoic acid derivative
4	3.642	299	153	Dihydroxybenzoic acid rhamnoside
5	4. 42	315	108, 153	Dihydroxybenzoic acid glucoside
6	5.243	371	135, 191	Caffeoylglucaric acid
7	5.862	169	125	Gallic acid
8	6.338	341	108, 167	Vanillyl quinic acid
9	7.124	153	108	3,4-dihydroxybenzoic acid
10	9.452	225	137	Hydroxybenzoic acid glycerol
11	10.921	353	135, 191	Chlorogenic acid
12	11.676	311	137	Hydroxybenzoic acid quinyl ester
13	12.289	329	153	Dihydroxybenzoic acid glucuronide
14	13.102	223	133,81	Pellitorine
15	15.075	181	107, 135	Dihydrocaffeic acid
16	17.482	367	191	Feruloylquinic acid
17	19.437	353	179, 191	Cryptochlorogenic acid
18	20.607	179	135	Caffeic acid
19	21.88	163	119	p-Coumaric acid
20	23.919	337	191	p-Coumaroylquinic acid
22	24.853	161	133	Hydroxycoumarin
22	27.796	515	179, 191	Isochlorogenic acid b
23	28.521	447	301	Quercetin rhamnoside
24	48.907	515	179, 191	Isochlorogenic acid C

Rt: retention time; **M.W**: molecular Weight; **MS/MS** Tandem Mass spectrometry

Table S2. Effects of Fentanyl and/or aqueous extract of *A. pyrethrum* (AEAP) on the abundance of the microbiota during the withdrawal phase.

	CFUX 10^6/ ML				
	Control	AEAP	Fentanyl	Fentanyl+post AEAP	Fentanyl+co AEAP
Phylum					
Firmicutes	4.34	12.2	2.13	0.19	4.31
Proteobacteria	0.11	1.62	0.00	0.00	0.00
Actinobacteria	0.04	0.29	0.00	0.05	0.05
Orders					
Lactobacillales	4.31	12.20	2.07	0.14	3.71
Bacillales	0.03	0.00	0.04	0.05	0.55
Mycobacteriales	0.00	0.00	0.02	0.00	0.00
Micrococcales	0.00	0.00	0.00	0.05	0.05
Pasteurellales	0.04	0.29	0.00	0.00	0.00
Enterobacterales	0.11	1.62	0.00	0.00	0.00
Family					
Lactobacillaceae	4.63	12.78	2.07	4.24	3.90
Staphylococcaceae	0.03	0.00	0.06	0.05	0.69
Streptococcaceae	0.20	0.58	0.00	0.00	0.19
Pasteurellacea	0.04	0.29	0.00	0.00	0.00
Enterobacteriaceae	0.18	1.62	0.00	0.00	0.00
Corynebacteriaceae	0.00	0.00	0.02	0.00	0.00
Microbactyeriaceae	0.00	0.00	0.00	0.05	0.00
Kitasatosporale	0.00	0.00	0.00	0.00	0.05
Species					
Ligilactobacillus faecis	0.93	9.26	1.76	0.00	2.32
Ligilactobacillus murinus	0.18	0.00	0.00	0.00	0.00
Lactobacillus gasseri	0.00	0.00	0.09	0.00	0.00
Lactobacillus sharpeae	0.00	0.00	0.00	2.05	0.00
Limosilactobacillus reutri	3.20	2.94	0.22	0.14	1.39
Staphylococcus hominis	0,03	0.00	0.00	0.00	0.09
Staphylococcus epidermidis	0.00	0.00	0.02	0.00	0.14
Staphylococcus pasteuri	0.00	0.00	0.04	0.05	0.46
Streptococcus anginosus	0.02	0.29	0.00	0.00	0.00
Streptococcus hyointestinalis	0.18	0.29	0.00	0.00	0.19
Haemophilus haemolyticus	0.04	0.29	0.00	0.00	0.00
Microbacterium arborescens	0.00	0.00	0.00	0.05	0.00
Streptomyces mutabilis	0.00	0.00	0.00	0.00	0.05
Corynebacterium stationis	0.00	0.00	0.02	0.00	0.00
Escherichia Coli	0.18	1.62	0.00	0.00	0.00
Total	4.76	14.69	2.15	2.29	4.69
